# Endoscopic ultrasound-guided cyanoacrylate injection for the treatment of gastric variceal bleeding after failed endoscopic embolization

**DOI:** 10.1055/a-2644-4314

**Published:** 2025-08-08

**Authors:** Lu Yang, Zhuang Kangmin

**Affiliations:** 1 665811 Department of Gastroenterology, Nanfang Hospital, Southern Medical University, Guangzhou, China

Gastric variceal (GV) hemorrhage is one of the most life-threatening adverse events in cirrhotic patients with portal hypertension, with an annual incidence of 5–15% 1 . Although less common than esophageal variceal bleeding, GV bleeding is associated with higher severity and mortality, reaching a 6-week mortality rate of 20% 2 . Endoscopic cyanoacrylate (CYA) injection remains the standard treatment for GV bleeding. However, failure occurs in 15–30% of cases due to factors such as obscured deep perforating veins, inadequate glue dispersion, or vascular anatomical variations 3 . Endoscopic ultrasound-guided selective variceal devascularization (EUS-SVD) has emerged as a salvage therapy, offering real-time visualization of vascular structures and hemodynamics. While EUS-SVD is primarily used for prophylactic management, this case demonstrates its efficacy in achieving hemostasis after failed conventional endoscopic CYA injection.

A 49-year-old male with a history of hepatitis C-related cirrhosis (18 years) and prior GV bleeding treated with CYA injection in 2016 presented with hematemesis and melena. Laboratory tests revealed thrombocytopenia (platelets: 64 × 109/L; reference: 125–350 × 109/L). Abdominal CT confirmed cirrhosis, splenomegaly, and splenic vein collaterals. Endoscopy showed severe GOV1 type varices (The titanium clip was left in place during a previous endoscopy treatment at an external hospital) ( Fig. 1 ). Under general anesthesia, the patient underwent endoscopic gastric variceal obturation (GVO). Persistent active bleeding from the target vein was observed during the procedure ( Video 1 ). Conversion to EUS-guided intervention was performed. EUS identified a gastric fundal variceal cluster (8 mm diameter). Using a 19-G needle (COOK), the variceal vein at the greater curvature was punctured under Doppler guidance to avoid perforators, and 1.0 mL of CYA was injected ( Fig. 2 , Video 1 ). Post-procedural EUS confirmed cessation of blood flow, and follow-up endoscopy demonstrated complete variceal obliteration ( Video 1 ). The patient experienced no complications (e.g., fever, embolism) and was discharged on day 3. No rebleeding occurred during the 6-month follow-up.

**Fig. 1:**
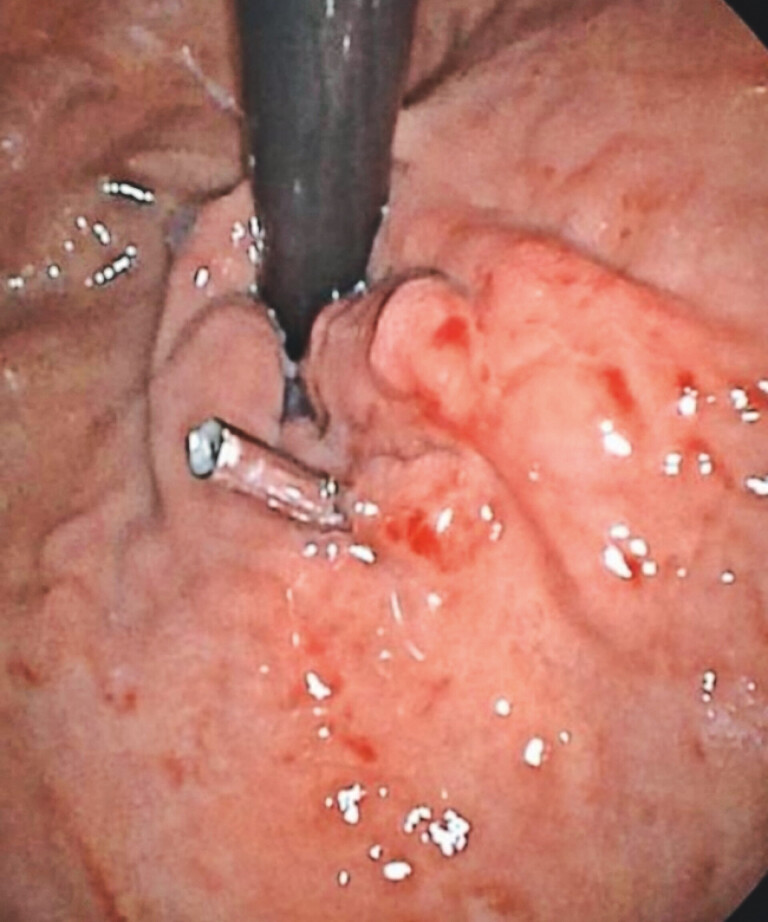
Endoscopic view of type 1 gastroesophageal varices in accordance with the Sarin classification.

**Fig. 2:**
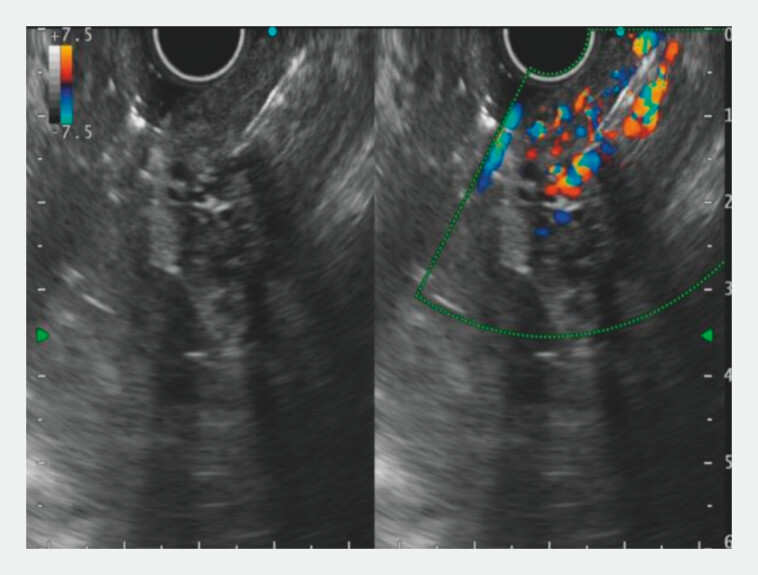
Endoscopic ultrasonography (EUS) with color Doppler evaluation of blood flow in gastric varices and EUS-guided injection of cyanoacrylate.

Endoscopic ultrasound (EUS)-guided intervention for active bleeding following endoscopic treatment.Video 1

GV bleeding remains a critical challenge in cirrhotic patients. While endoscopic CYA injection is first-line therapy, failure rates necessitate alternative approaches. Nonendoscopic options include transjugular intrahepatic portosystemic shunt and balloon-occluded retrograde transvenous obliteration 4 . However, advancements in endoscopic techniques, such as EUS-guided glue embolization (EUS-GE), offer minimally invasive solutions with high efficacy.

In this case, EUS-GE succeeded where conventional endoscopy failed, likely due to enhanced visualization of the variceal architecture, including the feeding artery (left gastric vein branch) and drainage vein (gastrorenal shunt). A retrospective study by Bhat et al. 5 (n = 152) reported a 98% immediate hemostasis rate and 8% 1-year rebleeding rate with EUS-GE, outperforming traditional GVO (25–35% rebleeding). Coil-assisted embolization was not utilized here due to the varices’ moderate caliber.

This case underscores its safety and feasibility in acute settings, aligning with literature supporting its role in managing complex variceal anatomy.

Endoscopy_UCTN_Code_TTT_1AO_2AD

## Notes

Endoscopy E-Videos https://eref.thieme.de/e-videos
*E-Videos* is an open access online section of the journal *Endoscopy* , reporting on interesting cases and new techniques in gastroenterological endoscopy. All papers include a high-quality video and are published with a Creative Commons CC-BY license. Endoscopy E-Videos qualify for HINARI discounts and waivers and eligibility is automatically checked during the submission process. We grant 100% waivers to articles whose corresponding authors are based in Group A countries and 50% waivers to those who are based in Group B countries as classified by Research4Life (see: https://www.research4life.org/access/eligibility/ ). This section has its own submission website at https://mc.manuscriptcentral.com/e-videos .
